# CBCT: knowledge, attitude, and practice among dentists

**DOI:** 10.1186/s12903-025-06870-x

**Published:** 2025-10-06

**Authors:** Matvey Uvarichev, Valeria Sherstneva, Anna Mikheikina, Maria Polyakova, Alexandr Zaytsev, Vladlena Doroshina, Inna Sokhova, Ksenia Babina, Nina Novozhilova

**Affiliations:** 1 Department of Therapeutic Dentistry, FSAEI HE I.M. Sechenov First MSMU of MOH of Russia (Sechenovskiy University), Moscow, 119991 Russia; 2 Institute of Foreign Languages for Professional Purposes, FSAEI HE I.M. Sechenov First MSMU of MOH of Russia (Sechenovskiy University), Moscow, 119991 Russia

**Keywords:** Dentistry, Cross-sectional survey, Practice patterns, Three-dimensional imaging

## Abstract

**Background:**

CBCT is an established diagnostic technique in oral medicine due to a relatively low dose and cost and high resolution. However, the use of CBCT should be restricted to situations where conventional dental radiography does not provide sufficient information.

**Methods:**

We aimed to assess the knowledge, attitude, and practice of Russian dentists towards the use of CBCT. A cross-sectional anonymous online survey was conducted among dental professionals of various specialties. The 15-minute survey consisted of four sections: demographic characteristics, knowledge (nine questions), attitude (five questions), and practice (eight questions). A Mann–Whitney U test was performed to compare these variables in the groups. Fisher’s exact test was used to compare the proportions. Spearman’s rank correlation coefficient was calculated to reveal pair-wise correlation between knowledge, attitude, and practice scores.

**Results:**

We analyzed 388 questionnaires. Most respondents showed a fair (42.5%) or poor (42.5%) knowledge of CBCT. We found a significant influence of the specialty and workplace (government-funded vs. private clinics) on the knowledge score. 90% of dentists demonstrated a positive attitude towards the use of CBCT. The attitude score was significantly influenced by the following factors: specialty, experience, gender, and workplace. While a vast majority of the respondents (92%) used CBCT in their practice, more than half of the participants (50.3%) never had training on CBCT performing and/or interpretation. The use of CBCT varied among different specialties, but no significant differences were found among dentists working at different workplaces and those with different clinical experience.

**Conclusions:**

The surveyed dentists had insufficient knowledge of CBCT, but commonly prescribed this imaging modality and demonstrated positive attitude towards it.

**Supplementary Information:**

The online version contains supplementary material available at 10.1186/s12903-025-06870-x.

## Introduction

Cone-beam computed tomography (CBCT) is a digital volume imaging technique which enables a three-dimensional visualization of anatomic structures in the dental, maxillofacial, and ear-nose-throat regions [1, 2]. It has become an established diagnostic technique in oral medicine due to a relatively low dose and cost and high resolution [3, 4]. CBCT provides a comprehensive set of cross-sectional images, horizontal, vertical, and axial views of structures [5], and a possibility of sectioning the volume in all planes without distortion and anatomic overlays [6–8]. CBCT possesses numerous advantages over other imaging techniques [9]. Compared to medical CT, CBCT is less expensive, less space-consuming and provides a reduced average radiation dosage and a possibility for adjusting the field of view (from the entire maxillofacial skeleton, i.e., large field of view, to a restricted dento-alveolar region involving a few teeth, i.e., small field of view) [10]. Compared to panoramic and intraoral imaging characterized by two-dimensional representation of 3-dimensional objects, CBCT allows multiplanar reconstruction, which is of particular importance for orthodontic, implant, and maxillofacial surgery planning, in periodontology, in clinical and surgical endodontics, and in the evaluation of the temporomandibular joint [6, 11–13]. Therefore, CBCT eliminates most disadvantages of periapical radiography and provides improved visualization of complex anatomy and high diagnostic accuracy [6, 7]. However, it is crucial to acknowledge the limitations of CBCT. First, it may have lower contrast and higher radiation dosages when compared to two-dimensional radiographic techniques [9]. Second, CBCT is inable to accurately represent soft tissues. Finally, various types of artifacts, mainly produced by metal restorations may hamper visualization of the region of interest on CBCT images and analysis of underlying structures [7, 14].

There is a growing body of literature which recognises that CBCT should be restricted to situations where its benefits outweigh the potential risks and conventional dental radiography does not provide sufficient information for formulating a diagnosis and treatment planning [15, 16]. A guiding principle of radiation safety is to maintain radiation doses as low as reasonably achievable (ALARA) [6, 17, 18]. In dentistry, a specified principle of “as low as *diagnostically acceptable*” (ALADA) was introduced. It combines the ALARA principle with the appropriate settings of the CBCT unit (tube current, voltage, resolution parameters) [18, 19]. All in all, the dentists’ decision on selection of an appropriate imaging technique should be based on careful assessment of indications for CBCT, as well as consideration of the ALARA and ALADA principles. This requires a human decision-making ability and a thorough understanding of the technology, its limitations, and relevance in each individual clinical case [18]. The decision should always be guided by the principle of doing no harm and ensuring that the benefits to the patient outweigh any potential risks or costs [6]. In a number of studies, it has been shown that in many fields of dentistry CBCT prescription was based on practitioners’ attitudes rather than on scientific evidence despite the availability of evidence-based recommendations. Several studies highlighted a lack of knowledge on CBCT [20–22] and radiation protection in general [23] among dental practitioners.

To the best of our knowledge, there are no published reports on the dentists’ awareness of evidence-based application of CBCT in Russia. At the same time there is a tendency towards unnecessary CBCT prescription to many patients, including children, which may increase individual and population radiation risks. Therefore, this study was designed to assess knowledge, attitudes, and practice of Russian dental practitioners towards the use of CBCT.

## Materials and methods

This cross-sectional open online survey was conducted between September 2023 and February 2024 after ethical approval (Protocol No. 16–23, September 14, 2023) was obtained from the Ethics Committee of Sechenov University, Moscow, Russia.

A questionnaire was developed specifically for this study in Russian based on the latest evidence-based literature on the topic [15, 24–29]. The expert panel (consisting of 3 subject experts in the field of dentistry and 2 subject experts in the field of radiology) approved the content validity of the questionnaire. A pilot test of the questionnaire was performed on a pilot sample excluded from the final analysis (*n* = 30). Feedback was collected concerning the order of questions and the time taken to complete the survey and was used to eliminate or rephrase ambiguous phrases. The finalized 15-minute survey consisted of 28 close-ended questions divided into four parts: basic demographic data (6 questions), knowledge (9 questions), attitude (5 questions), and practice (8 questions).

The knowledge-based items were three single-choice question, two multiple-choice questions, and four yes–no questions. Each correct answer was given a score of one. The item asking about the factors that influence the effective dose scored 1 for each correct answer; thus, the maximum score for the item was 4. The item asking about familiarity with guidelines and recommendations scored 1 if a participant chose at least one point from the list and scored 2 if a participant chose two points from the list or more. The maximum score for knowledge questions was 13. To obtain a total knowledge score, scores of all items were summed; the knowledge score was categorized as poor if it was lower than 50%, fair if it was between 50% and 75%, and good if it was higher than 75%.

In the attitude section, the respondents could agree or disagree with the statements using a five-point Likert scale. The overall score range in the section measuring attitudes was between 5 and 25. The overall attitude was regarded as positive if the score was higher than 50% or negative if the score was lower than 50%.

In the practice section, the participants were asked to mark the answer that best fits their practice on the use of CBCT.

We have collected the data through Google forms. The link was shared using different social media applications (professional groups in Telegram, WhatsApp, and VK). A convenience sampling method was used. The participation was voluntary; no identifying information was included. All the data were treated confidentially. Respondents were allowed to complete the questionnaire after they provided informed consent to participate in the survey and to have the results of the study published in a journal article. Providing answers to all the questions was mandatory, therefore only completed questionnaires were analyzed. The form required signing into Google account, thus limiting the participants to submit only one response.

The targeted samples included practitioners working in different fields of dentistry in Russia. The total number of the study population was estimated to be around 72,000 (a number of dental practitioners according to Federal State Statistics Service).

A minimum sample size of 383 respondents was calculated assuming a 95% CI and a margin of error of 5% using the following formula:

Sample size = $\:\:\frac{\frac{z^2\times\:p\left(1-p\right)}{e^2}}{1+\left(\frac{z^2\times\:p\left(1-p\right)}{e^2N}\right)}$, where.

z (z-score) = 1.96 (95% CI);

p (standard deviation) = 0.5;

e (a margin of error) = 0.05;

N (a population size) = 72000.

Data manipulation was performed through MS Excel (version 16.71 (23031200) Microsoft Corp., Mountain View, CA, USA) and R version 4.2.3 (2023-03-15), RDevelopment Core Team, Columbia university, New York, NY, USA) with the packages “doBy,” “rstatix,” and “stats” and using RStudio version (2023.03.0 + 386). The data were presented as means, medians, standard deviations, 25th and 75th percentiles (quantitative variables), and counts and percentages (qualitative variables). A Shapiro-Wilk test was used to assess the normality of distribution of the quantitative variables (knowledge, attitude, and practice overall scores), and a Mann–Whitney U test was performed to compare these variables in the groups. Fisher’s exact test was used to compare the proportions. Spearman’s rank correlation coefficient was calculated to reveal pair-wise correlation between knowledge, attitude, and practice scores.

## Results

Of the 388 respondents, 141 (36.3%) were men and 247 (63.7%) were women. A majority of the respondents were aged between 20 and 30 years (*n* = 217, 55.9%). Table 1 shows participants’ demographic characteristics, knowledge, and attitudes towards the use of CBCT in clinical practice depending on their gender, age, specialty, clinical experience, and workplace.

**Table 1:** Knowledge and attitudes towards the use of CBCT in clinical practice among participants with different clinical experience and of different specialties, working in the private or government-funded sectors

Sociodemographic data		Knowledge (max 13)		Attitude (max 25)	
	*n* (%)	Mean (sd)	Median (IQR)	Mean (sd)	Median (IQR)
Total		6.8 (2.2)	7 (5; 8)	18.4 (4.2)	19 (16; 21)
Gender					
Male	141 (36.3)	6.7 (2.3)	7.0 (5.0; 8.0)	18.0 (4.4)	18 (15; 21)
Female	247 (63.7)	7.1 (2.1)	7.0 (6.0; 9.0)	19.0 (3.7)	19 (17; 21)
		DFn = 1, Dfd = 386, F = 2.542, *p* = 0.112		DFn = 1, Dfd = 386, F = 5.391, *p* = 0.021*	
Age					
20–30	217 (55.9)	7.0 (2.1)	7.0 (6.0; 8.0)	18.5 (3.9)	19 (16; 21)
30–40	116 (29.9)	6.8 (2.3)	7.0 (5.0; 8.0)	18.5 (4.6)	19 (15.75; 22)
40–50	46 (11.9)	6.4 (2.4)	6.0 (6.0; 8.0)	17.5 (4.2)	17.5 (14.25; 20)
50+	9 (2.3)	6.7 (3.2)	4.0 (4.0; 10.0)	18.3 (4.7)	19 (16; 21)
		DFn = 3, Dfd = 384, F = 0.876, *p* = 0.454		DFn = 3, Dfd = 384, F = 0.867, *p* = 0.458	
Specialty					
Conservative dentist	142 (36.6)	6.3 (2.3)^a^	6.5 (5; 8)	17.3 (4.7)^a^	17 (14; 21)
Pediatric dentist	21 (5.4)	7.0 (1.9)^a^	7 (6; 8)	16.8 (4.8)^a^	17 (13; 21)
Dental surgeon	42 (10.8)	7.4 (1.7)^a^	8 (6; 8.75)	19.4 (3.4)^b^	19 (17.25; 21)
Orthodontist	42 (10.8)	7.3 (2.6)^a^	8 (5.25; 9)	19.5 (4.1)^b^	21 (18; 22)
Prosthetic dentist	47 (12.1)	7.2 (1.8)^a^	7 (6; 8.5)	19.6 (2.5)^b^	20 (18; 21)
General dentist	70 (18.0)	6.9 (2.4)^a^	7 (5.25; 9)	19.2 (4.2)^b^	20 (17; 22)
Maxillo-facial surgeon	24 (6.2)	7.1 (1.8)^a^	7.5 (6; 8)	17.9 (2.6)^ab^	17.5 (17; 20)
		DFn = 6,Dfd = 381, F = 2.242, *p* = 0.039*		DFn = 6, Dfd = 381, F = 4.316, *p* < 0.001*	
Years of clinical experience					
< 2	124 (31.9)	6.9 (2.1)	7.0 (5.0; 8.0)	18.3 (3.6)^a^	18 (16; 21)
2–5	91 (25.8)	7.0 (1.9)	7.0 (6.0; 8.0)	18.5 (4.1)^a^	19 (16; 21)
6–10	57 (14.7)	7.3 (2.0)	8.0 (5.0; 9.0)	19.8 (3.7)^a^	21 (17; 23)
11–15	60 (15.5)	6.5 (2.6)	7.0 (4.0; 8.0)	17.9 (5.2)^a^	19 (14; 22)
15+	56 (14.4)	6.5 (2.6)	6.5 (5.5; 8.0)	17.9 (4.5)^a^	18 (15; 21)
		DFn = 5, Dfd = 382, F = 1.935, *p* = 0.088		DFn = 5, Dfd = 382, F = 2.386, *p* = 0.038*	
Clinic					
Private	73 (18.8)	7.0 (2.2)	7.0 (5.0; 9.0)	18.6 (4.0)	19 (16; 21.5)
Government-funded	315 (81.2)	6.3 (2.3)	7.0 (4.0; 8.0)	17.4 (4.9)	17 (14; 21)
		DFn = 1, Dfd = 386, F = 5.321, *p* = 0.022*		DFn = 1, Dfd = 386, F = 5.374, *p* = 0.021*	
Federal District					
Central	226 (58.2)	7.0 (2.4)	7.0 (6.0; 9.0)	18.2 (4.4)	18 (16; 21)
Northwestern	60 (15.5)	6.7 (2.1)	7.0 (5.0; 8.0)	19.2 (4.0)	19.5 (18; 22)
Volga	41 (10.6)	6.6 (1.8)	7.0 (5.0; 8.0)	18.5 (3.6)	19 (15; 22)
Siberian	25 (6.4)	6.5 (2.4)	6.0 (5.0; 8.0)	18.4 (4.5)	17 (14; 22)
North Caucasian	4 (1)	6.5 (1.7)	6.5 (5.0; 8.0)	18.5 (2.9)	18.5 (16; 21)
Southern	8 (2.1)	7.1 (1.3)	7.0 (6.0; 7.5)	18.2 (2.3)	17 (16.75; 21)
Ural	17 (4.4)	6.6 (1.9)	7.0 (6.0; 8.0)	17.5 (3.9)	16 (15; 17)
Far Eastern	7 (1.8)	6.6 (1.4)	6.0 (5.5; 8.0)	19.0 (1.9)	18 (17.5; 21)
		DFn = 6, Dfd = 380, F = 0.323, *p* = 0.943		DFn = 6, Dfd = 380, F = 0.510, *p* = 0.827	

*DFn D*egree of freedom for the numerator of the F ratio, * DFd *Degree of freedom for the denominator of the F ratio, *IQR* Interquartile range

### Knowledge

The mean knowledge score was 6.8 ± 2.2 and the median knowledge score was 7 (5; 8) (out of 13). Considering the cut-off points 0–7, 7–9 and 9–13 as poor, fair, and good, respectively, we found that 15% (*n* = 58) had good knowledge, 42.5% (*n* = 165) had fair knowledge and 42.5% (*n* = 165) had poor knowledge.

The ANOVA revealed a significant influence of the specialty (F(6, 381) = 2.24, *p* = 0.039) and workplace (F(1, 386) = 5.321, *p* = 0.022) on the knowledge score of the participants, while other factors (experience, gender, age, and federal district) had no impact on this parameter. Table 2 shows the distribution of responses to knowledge questions about the use of CBCT in clinical practice among dental professionals.

**Table 2:** Distribution of responses to knowledge questions about the use of CBCT in clinical practice among dental professionals, n (%)

Item	Variants	Answers *n* (%)
Can you explain the meaning of the ALARA principle?	Yes	74 (19.1)
	No	314 (80.9)
Can you explain the meaning of the ALADA principle?	Yes	48 (12.4)
	No	340 (87.6)
Which of the following guidelines/recommendations you are familiar with?	National guidelines (SanPiN 2.6.1.1192-03) [39]	273 (70.4)
	AAE&AAOMR (endodontics) [22]	30 (7.7)
	SEDENTEXCT [18]	9 (2.3)
	ADA CSA (dentistry) [32]	6 (1.5)
	AAOMR (orthodontics) [20]	9 (2.3)
	AAOMR (implantology) [23]	4 (1.0)
	None	96 (24.7)
Smaller voxel size results in…	increased image quality and increased effective dose	202 (52.0)
	increased image quality and decreased effective dose	62 (16.0)
	decreased image quality and increased effective dose	4 (1.0)
	decreased image quality and decreased effective dose	21 (5.4)
	I don’t know	99 (25.5)
Compared with the effective dose of OPG, the effective dose of CBCT is…	several times higher	151 (38.9)
	somewhat higher	93 (24.0)
	somewhat lower	33 (8.5)
	several times lower	33 (8.5)
	I don’t know	78 (20.1)
Compared with the effective dose of spiral CT, the effective dose of CBCT is…	several times higher	28 (7.2)
	somewhat higher	41 (10.6)
	somewhat lower	76 (19.5)
	several times lower	98 (25.3)
	I don’t know	145 (37.4)
The effective dose of CBCT depends on…	voxel size	260 (67.0)
	field of view	235 (60.6)
	tube current	43 (11.1)
	patient’s age	34 (8.8)
	I don’t know	44 (11.3)
Is there any threshold below which the effective dose of CBCT is absolutely safe for the patient?	Yes	211 (54.4)
	No	177 (45.6)
Is it necessary to prescribe CBCT if the diagnosis and treatment strategy are clear based on the clinical examination and a two-dimensional x-ray?	Yes	154 (39.7)
	No	234 (60.3)

*AAE* American Association of Endodontists, *AAOMR* American Academy of Oral and Maxillofacial Radiology, *ADA CSA* American Dental Association Council on Scientific Affairs, *SEDENTEXCT* Safety and efficacy of a new and emerging dental X-ray modality, *SanPiN* Sanitary regulations and norms

A large majority of the participants were unable to explain the meaning of the ALARA and ALADA principles (81% and 88%, respectively). However, 70% (*n* = 273) of the respondents stated that they were aware of the national Sanitary Regulations and Norms on Radiation Protection (SanPiN 2.6.1.1192-03). Only 6 participants (1.5%) were familiar with an advisory statement on the use of CBCT in dentistry, while 24.7% (*n* = 96) were not familiar with any regulations or position papers on the topic.

Slightly more than half of the dentists (54.4%, *n* = 211) believed that there existed a radiation dose of CBCT absolutely harmless to the patients. Moreover, 40% (*n* = 154) of the respondents stated that CBCT should be performed even if there were sufficient clinical and radiographic data to make an accurate diagnosis.

### Attitude

The mean attitude score was 18.4 ± 4.2 (out of 25). 90% of dentists (*n* = 351) demonstrated a positive attitude towards the use of CBCT. The attitude score was significantly influenced by the following factors: specialty (*p* = 0.000316), experience (*p* = 0.038), gender (*p* = 0.021), and workplace (*p* = 0.021). Conservative (*p* = 17.3) and paediatric dentists (*p* = 16.8) had significantly lower attitude scores as compared to other specialties (Table 1).

A high percentage of dentists (88.9%, *n* = 336) believed (agreed or strongly agreed) that CBCT was the most informative radiographic method in their field (Table 3). More than half of the participants preferred CBCT as the imaging modality of choice. Two hundred and seventeen respondents (56.0%) held the view that CBCT was absolutely safe for the patients and only 72 (18.5%) disagreed with this.

**Table 3:** Distribution of responses to attitude questions about the use of CBCT in clinical practice among dental professionals, n (%)

Item	Respondents’ answers *n* (%)				
	SA	A	N	D	SD
CBCT is the most informative radiographic method in my field	266 (68.6)	70 (20.3)	30 (7.7)	4 (1.0)	9 (2.3)
I prefer CBCT to other radiographic methods	219 (56.4)	50 (12.9)	79 (20.4)	23 (5.9)	17 (4.4)
CBCT should be used as a screening method before the clinical examination	120 (30.9)	51 (13.1)	78 (20.1)	51 (13.1)	88 (22.7)
CBCT should be used only if other methods failed to ascertain the diagnosis	104 (26.8)	52 (13.4)	84 (21.6)	66 (17.0)	82 (21.1)
The radiation exposure associated with CBCT is absolutely safe	150 (38.7)	67 (17.3)	99 (25.5)	37 (9.5)	35 (9.0)

*CBCT* Cone-beam computed tomography, *SA* Strongly agree, *A* Agree, *N* Neutral, *D* Disagree, *SD* Strongly disagree

Correlation analysis revealed a strong positive correlation (rho = 0.82, *p* < 0.0001) between the knowledge scores and attitude scores of the participants.

### Practice

More than 70% of the participants (*n* = 279) had CBCT units at their workplaces; however, dentists did not perform CBCT themselves [Table 4]. The availability of CBCT did not differ between the private (73%) and government-funded (72%) sectors (*p* = 1.0).

**Table 4:** Distribution of responses to questions about the use of CBCT in clinical practice among dental professionals, n (%)

Item	Variants	Answers *n* (%)
Do you have a CBCT unit at your workplace?	Yes	279 (71.9)
	No	109 (28.1)
Who is responsible for performing CBCT at your workplace?	Dentist	14 (3.6)
	Another person	265 (68.3)
Which protective equipment is used for CBCT acquisition at your workplace?	Lead apron	269 (69.3)
	Thyroid collar	120 (30.9)
	X-ray shielding screen	164 (42.3)
Do you use CBCT in your practice?	Yes	357 (92.0)
	No	31 (8.0)
Have you ever had training on CBCT acquisition and/or interpretation?	Yes	193 (49.7)
	No	195 (50.3)
How often do you prescribe CBCT?	To almost every patient	92 (23.7)
	To a majority of my patients	106 (27.3)
	To around half of my patients	78 (20.1)
	To a small number of patients	81 (20.9)
	Never	31 (8.0)
What factors do you consider when deciding to prescribe CBCT?	Clinical situation	331 (85.3)
	Potential pregnancy	286 (73.7)
	Malignant comorbidities	190 (49.0)
	Previous radiation exposure	78 (20.1)
	Presence of metall constructions in the RoI	139 (35.8)
	Age of the patient	2 (0.5)
Which FoV would you prefer if the RoI includes 1–2 teeth and there is no evidence of other pathological processes in other areas?	Small	211 (54.4)
	Large	146 (37.6)
	Don’t use CBCT	31 (8.0)

*CBCT * Cone-beam computed tomography, *RoI* Region od interest, *FoV* Field of view

**Fig. 1:**
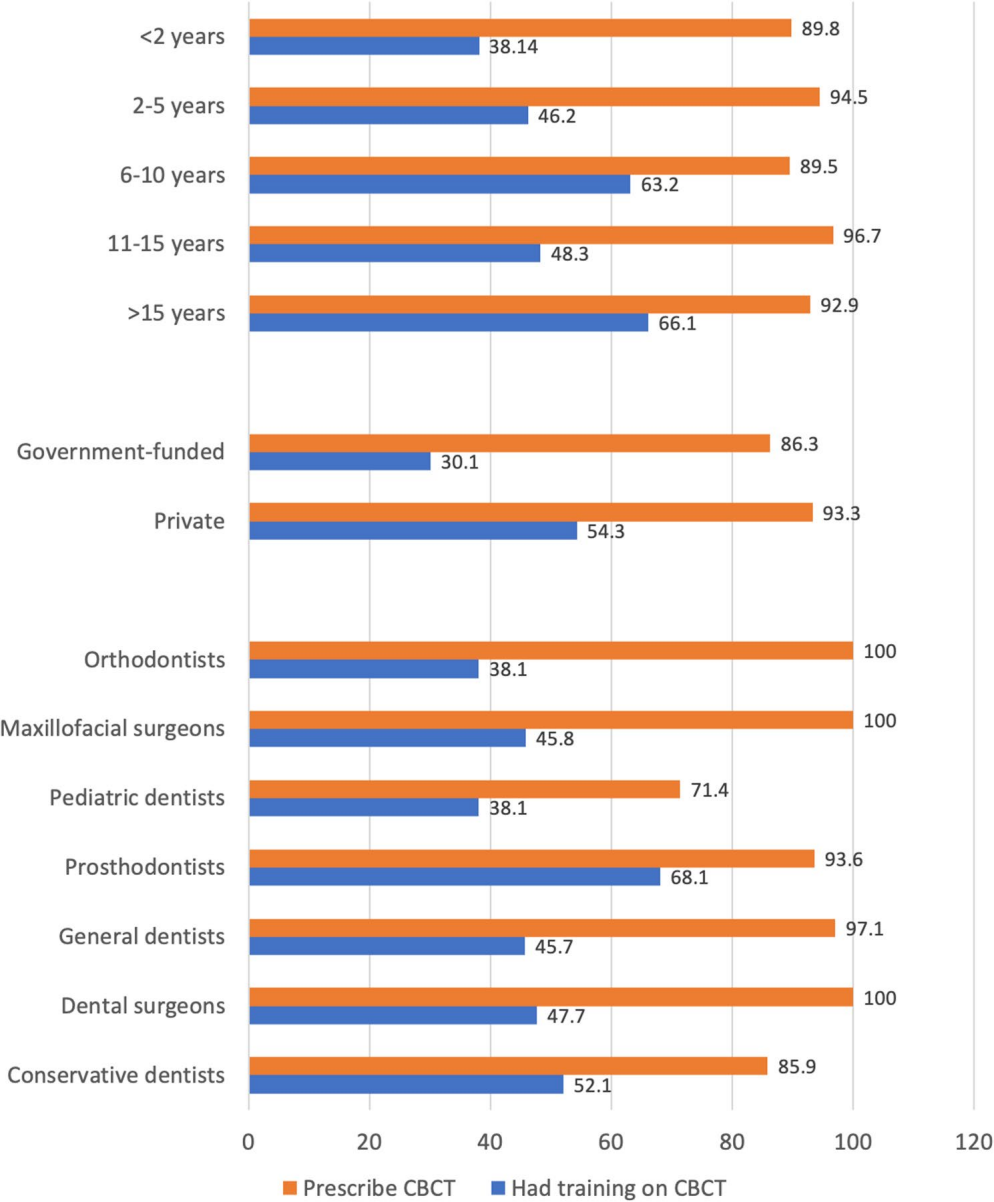
The use of CBCT in practice and completion of training programs on CBCT among the participants with different clinical experience and of different specialties, working in the private or government-funded sectors.

While a vast majority of the respondents (92.0%, *n* = 357) used CBCT in their practice, more than half of the participants (50.3%, *n* = 195) never had training on CBCT performing and/or interpretation. The use of CBCT varied among different specialties (*p* = 0.0004998), while no significant differences were found among dentists working at different workplaces (*p* = 0.05562) and those with different clinical experience (*p* = 0.4268) [Figure 1].

**Fig. 2:**
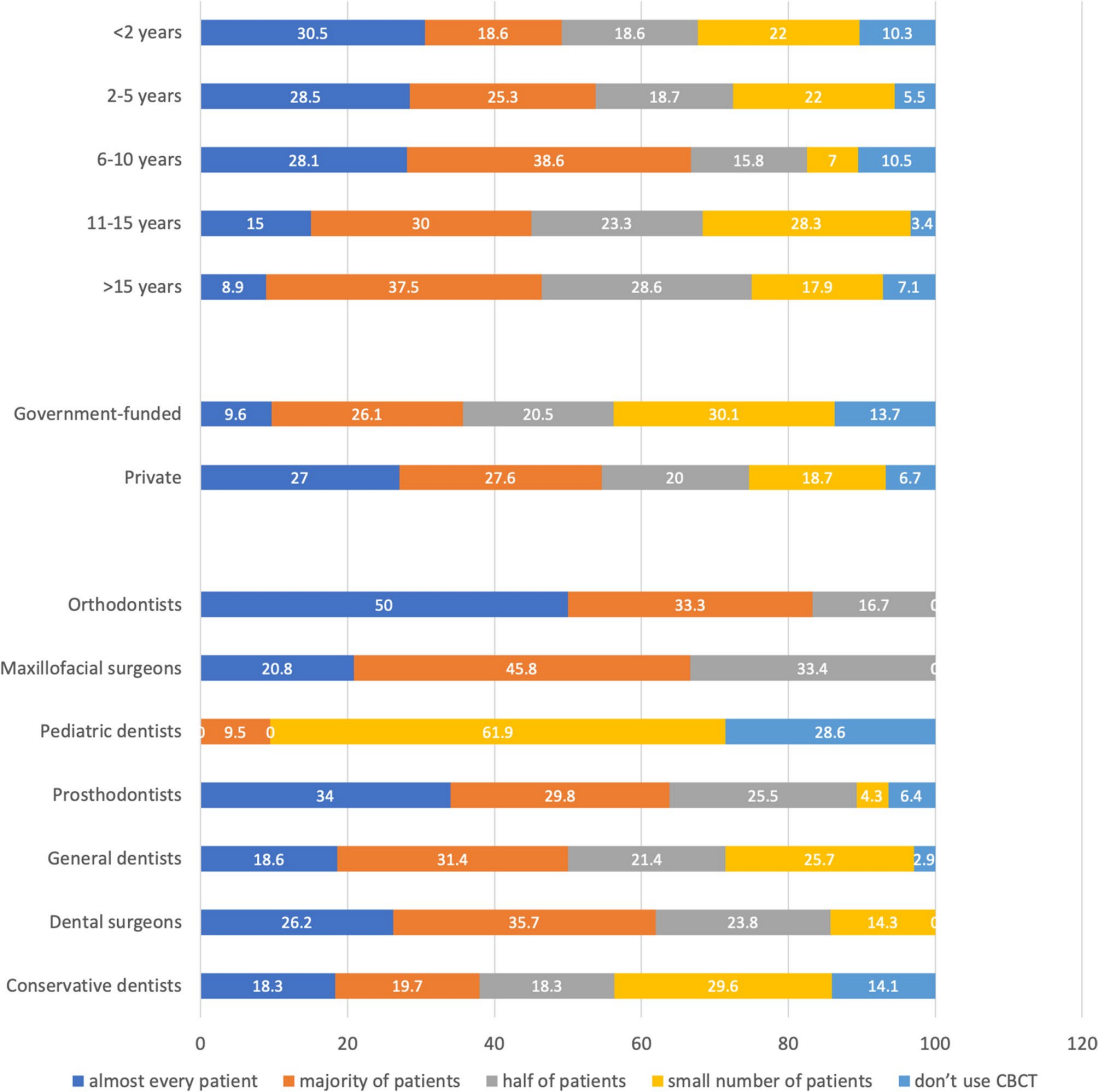
Frequency of CBCT prescription among the participants with different clinical experience and of different specialties, working in the private or government-funded sectors.

Over 60% of maxillofacial and dental surgeons and prosthetic dentists and 83% of orthodontists prescribed CBCT to a majority of their patients/to almost every patient. On the other hand, 62% of paediatric dentists prescribed this imaging modality to a small number of their patients (*p* < 0.001). Besides, dentists working in private sector used CBCT significantly more often than those working in the government-funded sector (*p* < 0.001). The percentage of dental professionals prescribing this imaging modality to almost every patient gradually decreased with the increase of the years of clinical experience (*p* < 0.001) [Figure 2].

## Discussion

In our study, we aimed to assess the knowledge, attitudes, and practice of dental practitioners towards the use of CBCT. A large majority of the respondents demonstrated poor knowledge and positive attitudes towards CBCT. We found a strong positive correlation between the knowledge scores and attitude scores of the participants.

This study revealed insufficient awareness of the dentists regarding CBCT. Similar results have been reported in previous studies. Masyte et al. expressed concerns regarding the knowledge of Lithuanian dentists about exposure factors and CBCT performance for pediatric patients [21]. Non-satisfactory knowledge of Indian dental practitioners on the use of CBCT has been reported by Chinna [20]. A study by Wazir et al. revealed a mean knowledge on radiation protection to be 60% [30]. In a study by Yalcinkaya et al., the knowledge of endodontists depended on their age: specialists aged 40 and above had a significantly lower knowledge of CBCT compared to younger groups [31]. Wazir et al. reported that this parameter was gradually increasing with clinical experience [30]. In our study, the specialty and workplace had a significant impact on the knowledge scores of the participants, while their experience, gender, and age had no impact on this parameter.

According to our results, 70% of the respondents were aware of the national guidelines. It should be noted that this document deals with radiation protection in general and does not provide specific regulations on CBCT. Almost 25% were not familiar with any regulations or position papers on the topic at all. This may be due to the fact that the majority of guidelines and position papers are in English and have not been translated to Russian, which limits the access of non-English-speaking dentists. A survey of Shanmugam et al. showed that 52.5% of undergraduates and 94.5% of house surgeons were aware of the national and international guidelines regarding radiation doses [32]. Parveen reported that 49% of the respondents were aware of guidelines for the use of CBCT in orthodontics [33]. In other studies, the level of awareness was even lower [23, 30]. The variations in the level of guidelines awareness may be explained by a self-reported nature of the survey, and the results should be interpreted with caution.

The percentage of the participants of our survey with knowledge of the “As Low As Diagnostically Acceptable” (ALADA) principle (12%) was lower than the percentage of the participants with knowledge of “As Low As Reasonably Achievable” (ALARA) principle (19%). Similarly, in a study by Parveen et al. the orthodontists and orthodontic residents were less commonly aware of the ALADA principle (28.9%) compared with ALARA (53.5%) [33]. Alnuaimy et al. surveyed general dentists and specialized dentists and found that they knew ALARA or ALADA in 41.3% and 61% of cases, respectively [34]. Other surveys demonstrated that there was a great deal of variation in the respondents’ answers regarding the familiarity with the ALARA principle (13.7–98.6% depending on the professional experience and workplace) [23, 30, 33].

Almost two-thirds of the survey participants knew that an effective dose of CBCT depends on the field of view and voxel size of the scan. Yalda et al. reported low level of awareness on the exposure settings (kV, mAs) used during CBCT acquisition in their practice [35]. However, in some of the previous surveys, the majority of dentists could specify the technical parameters they used [36]. Alarmingly, in our study more than half of the dentists believed that there existed a radiation dose absolutely harmless to patients. Moreover, 56% of our participants believed that CBCT is absolutely safe for the patients and only 19% disagreed with this point of view. Similarly, in a survey by Almohaimede et al. only around 60% of the respondents knew that dental X-rays are harmful and can lead to DNA alterations [23]. Other studies revealed that between 2% and 24% of the dental professionals believed that dental X-rays are harmless [30, 32]. Our survey also revealed a low number of the participants who correctly identified differences in effective doses of CBCT and panoramic imaging (39%) and CBCT and spiral CT (25%). Similarly, in a study by Lavaya et al., a majority of the respondents were unsure about the radiation exposure for CBCT as compared to other types of imaging [22].

Regarding the dentists’ opinion about CBCT, a vast majority of the respondents demonstrated positive attitude. These findings corroborate the results reported by Hol [37]. Moreover, 40% of the respondents believed that CBCT should be performed even if there were sufficient clinical and radiographic data to state an accurate diagnosis; 44% of the participants stated that CBCT should be used as a screening method before clinical examination. This comes into conflict with the existing guidelines which state that CBCT does not present sufficient evidence to be indicated as the exam of first choice and should be used as a second-level test [8, 24, 26, 28, 29, 38].

Regarding CBCT availability, more than 70% of the participants had CBCT machines at their workplaces. In a study by Mathew et al., 53% of the participants had a CBCT unit located at their place of clinical practice [39]. Other studies have reported lower availability of CBCT systems in their practices (28% [21], 9.5% [40]).

In our study, the use of CBCT was reported by 92% of the respondents. The lowest rate was among pediatric dentists (71%), while 100% of orthodontists and dental and maxillofacial surgeons reported the use CBCT in their practice. Previous studies have reported less frequent use of CBCT [21, 40]. Masyte et al. found that CBCT imaging was most commonly performed by prosthodontists (63%), oral and maxillofacial surgeons (59%), and orthodontists (57%), while pediatric dentists (19%) and general practitioners (17%) referred to CBCT less commonly [21]. In a study by Gillies et al., the highest frequency of CBCT was reported for implant treatment planning (31% of the respondents performed CBCT in every case, while 67% in some cases); the use of CBCT was less frequent in cases of endodontic treatment, teeth extractions, and orthodontic treatment [40]. In our study, prosthetic dentists, orthodontists, and maxillofacial and dental surgeons prescribed CBCT more frequently than dentists of other specialties, while a majority of paediatric dentists prescribed CBCT to a small number of their patients.

Despite the frequent use of CBCT in their practice, more than half of the participants of our survey never had training on how to perform CBCT and/or how to interpret CBCT results. According to Ersan et al., most of the current dentists have received insufficient or no training in the application and interpretation of 3D images [41]. Gillies found that 21% had never taken courses on CBCT [40]. Previous studies have also shown insufficient training on CBCT during university education [10, 42].

According to our results, almost 40% of the respondents preferred to use large-FoV CBCT even if the region of interest was limited to 1 or 2 teeth. In a study by Gillies et al., a majority of the respondents (72%) most commonly used FoV with diameters between 5 cm and 8 cm [40]. Other studies have reported that smallest FoV was the preferred one [36, 37, 39, 43]. The use of smallest possible FoV is justified by guidelines [15, 24, 25, 28, 29, 33] and can improve image quality due to the decrease in X-ray scatter and smaller volume of tissues to be scanned [35].

A minority of the practitioners in our study considered the ages of the patients, although the frequency of CBCT prescription was the lowest among paediatric dentists. In a study by Yalda et al., a majority of dental practitioners (92%) did not perform CBCT in children [35]. Similar results were reported in the study by Alzamzami et al. who found that only 9.2% of the respondents used CBCT in paediatric patients [43]. Taking into consideration high effective dose of CBCT and a greater risk of stochastic effects in children and young people, it is unsurprising that the use of CBCT was lower in this age group. Also, 74% of dental practitioners considered potential pregnancy when prescribing CBCT. Previous studies have reported a cautious attitude of dental practitioners towards radiographic examinations in pregnant patients [30, 43]. Alzamzami et al. found that a vast majority of the respondents did not consider prescribing CBCT to pregnant women [43].

All in all, the results of the survey revealed alarmingly low knowledge on the CBCT use among dentists that results in CBCT overuse, posing a risk to patients’ safety. Therefore, it is necessary to include more information on the topic in the undergraduate curricula as well as develop advanced postgraduate courses based on the current guidelines and regulations on the use of CBCT in dentistry.

We readily acknowledge several limitations to our study. Since the survey was conducted online, it could have not reached dental practitioners who were not active internet users, specifically in rural areas. The self-reporting method of data collection could be a source of response bias. Also, as we used convenience sampling, some areas and dental specialties could be underrepresented in the study sample. Further in-depth research is required regarding the use of CBCT in different fields of dentistry.

## Conclusion

Based on our survey, Russian dentists have insufficient knowledge about CBCT but commonly prescribe this imaging modality and demonstrate positive attitude towards it. We also revealed a lack of training in performing CBCT and interpretating CBCT results among dental practitioners. The results obtained raise concerns regarding the inappropriate levels of CBCT use and underestimation of the risks associated with radiation exposure.

## Supplementary Information

Supplementary Material 1.

## Data Availability

The datasets used and/or analysed during the current study are available from the corresponding author on reasonable request.

## Abbreviations

CBCT: Cone-beam computed tomography
ANOVA: Analysis of variance
ALARA: As low as reasonably achievable
ALADA: As low as diagnostically acceptable
