# Supplementary material for: CBCT: knowledge, attitude, and practice among dentists
Source: BMC Oral Health. 2025 Oct 6;25:1540. doi: 10.1186/s12903-025-06870-x (PMC12502351; doi:10.1186/s12903-025-06870-x)
Supplement: Supplementary file 1 — Supplementary Material 1. [file 12903_2025_6870_MOESM1_ESM.docx]

| **Personal information** | |
| --- | --- |
| **1. What is your gender?** | - Male - Female |
| **2. What is your age?** | - 20-30 - 30-40 - 40-50 - >50 |
| **3. What is your specialty?** | - Conservative dentist - Pediatric dentist - Dental surgeon - Orthodontist - Prosthetic dentist - General dentist - Maxillo-facial surgeon |
| **4. How many years of clinical experience do you have?** | - < 2 - 2-5 - 6-10 - 11-15 - >15 |
| **5. What type of clinic are you employed in?** | - Government-funded - Private |
| **6. What Federal District do you work in?** | - Central - Northwestern - Volga - Siberian - North Caucasian - Southern - Ural - Far Eastern |

| **Knowledge** | |
| --- | --- |
| **K1: Can you explain the meaning of the ALARA principle?** | |
| - Yes | 1 |
| - No | 0 |
| **K2: Can you explain the meaning of the ALADA principle?** | |
| - Yes | 1 |
| - No | 0 |
| **K3: Which of the following guidelines/recommendations you are familiar with? Select all that apply.** | |
| - National guidelines (SanPiN 2.6.1.1192-03) | 1 |
| - AAE&AAOMR (endodontics) | 1 |
| - SEDENTEXCT | 1 |
| - ADA CSA (dentistry) | 1 |
| - AAOMR (orthodontics) | 1 |
| - AAOMR (implantology) | 1 |
| - None | 0 |
| **K4: Smaller voxel size results in…** | |
| - increased image quality and increased effective dose | 1 |
| - increased image quality and decreased effective dose | 0 |
| - decreased image quality and increased effective dose | 0 |
| - decreased image quality and decreased effective dose | 0 |
| - I don’t know | 0 |
| **K5: Compared with the effective dose of OPG, the effective dose of CBCT is…** | |
| - several times higher | 1 |
| - somewhat higher | 0 |
| - somewhat lower | 0 |
| - several times lower | 0 |
| - I don’t know | 0 |
| **K6: Compared with the effective dose of spiral CT, the effective dose of CBCT is…** | |
| - several times higher | 0 |
| - somewhat higher | 0 |
| - somewhat lower | 0 |
| - several times lower | 1 |
| **K7: The effective dose of CBCT depends on…** | |
| - voxel size | 1 |
| - field of view | 1 |
| - tube current | 1 |
| - patient’s age | 1 |
| - I don’t know | 0 |
| **K8: Is there any threshold below which the effective dose of CBCT is absolutely safe for the patient?** | |
| - Yes | 0 |
| - No | 1 |
| **K9: Is it necessary to prescribe CBCT if the diagnosis and treatment strategy are clear based on the clinical examination and a two-dimensional x-ray?** | |
| - Yes | 0 |
| - No | 1 |

| **Attitude** | |
| --- | --- |
| **A1: CBCT is the most informative radiographic method in my field:** | |
| Strongly disagree | 1 |
| Disagree | 2 |
| Neutral | 3 |
| Agree | 4 |
| Strongly agree | 5 |
| **A2: I prefer CBCT to other radiographic methods:** | |
| Strongly disagree | 1 |
| Disagree | 2 |
| Neutral | 3 |
| Agree | 4 |
| Strongly agree | 5 |
| **A3: CBCT should be used as a screening method before the clinical examination:** | |
| Strongly disagree | 1 |
| Agree | 2 |
| Neutral | 3 |
| Disagree | 4 |
| Strongly agree | 5 |
| **A4: CBCT should be used only if other methods failed to ascertain the diagnosis:** | |
| Strongly agree | 1 |
| Disagree | 2 |
| Neutral | 3 |
| Agree | 4 |
| Strongly disagree | 5 |
| **A5: The radiation exposure associated with CBCT is absolutely safe:** | |
| Strongly disagree | 1 |
| Disagree | 2 |
| Neutral | 3 |
| Agree | 4 |
| Strongly agree | 5 |

| **Practice** |
| --- |
| **P1: Do you have a CBCT unit at your workplace?** |
| - Yes |
| - No |
| **P2: Who is responsible for performing CBCT at your workplace?** |
| - dentist |
| - another person |
| **P3: Which protective equipment is used for CBCT acquisition at your workplace?** |
| - Lead apron |
| - Thyroid collar |
| - X-ray shielding screen |
| **P4: Do you use CBCT in your practice?** |
| - Yes |
| - No |
| **P5: Have you ever had training on CBCT acquisition and/or interpretation?** |
| - Yes |
| - No |
| **P6: How often do you prescribe CBCT?** |
| - To almost every patient |
| - To a majority of my patients |
| - To around half of my patients |
| - To a small number of patients |
| - Never |
| **P7: What factors do you consider when deciding to prescribe CBCT?** |
| - Clinical situation |
| - Potential pregnancy |
| - Malignant comorbidities |
| - Previous radiation exposure |
| - Presence of metall constructions in the RoI |
| - Age of the patient |
| **P8: Which FoV would you prefer if the RoI includes 1-2 teeth and there is no evidence of other pathological processes in other areas?** |
| - Small |
| - Large |
| - Don’t use CBCT |
